# Biventricular shape modes discriminate pulmonary valve replacement in tetralogy of Fallot better than imaging indices

**DOI:** 10.1038/s41598-023-28358-w

**Published:** 2023-02-09

**Authors:** Sachin Govil, Charlène Mauger, Sanjeet Hegde, Christopher J. Occleshaw, Xiaoyang Yu, James C. Perry, Alistair A. Young, Jeffrey H. Omens, Andrew D. McCulloch

**Affiliations:** 1 Department of Bioengineering, University of California San Diego, 9500 Gilman Drive, MC 0412, La Jolla, CA 92093-0412 USA; 2 Department of Anatomy and Medical Imaging, University of Auckland, Auckland, New Zealand; 3 Auckland Bioengineering Institute, University of Auckland, Auckland, New Zealand; 4 Department of Pediatrics, University of California San Diego, La Jolla, CA USA; 5 Division of Cardiology, Rady Children’s Hospital San Diego, San Diego, CA USA; 6 Department of Cardiology, Auckland District Health Board, Auckland, New Zealand; 7 Department of Biomedical Engineering, King’s College London, London, UK

**Keywords:** Congenital heart defects, Biomedical engineering

## Abstract

Current indications for pulmonary valve replacement (PVR) in repaired tetralogy of Fallot (rTOF) rely on cardiovascular magnetic resonance (CMR) image-based indices but are inconsistently applied, lead to mixed outcomes, and remain debated. This study aimed to test the hypothesis that specific markers of biventricular shape may discriminate differences between rTOF patients who did and did not require subsequent PVR better than standard imaging indices. In this cross-sectional retrospective study, biventricular shape models were customized to CMR images from 84 rTOF patients. A statistical atlas of end-diastolic shape was constructed using principal component analysis. Multivariate regression was used to quantify shape mode and imaging index associations with subsequent intervention status (PVR, n = 48 vs. No-PVR, n = 36), while accounting for confounders. Clustering analysis was used to test the ability of the most significant shape modes and imaging indices to discriminate PVR status as evaluated by a Matthews correlation coefficient (MCC). Geometric strain analysis was also conducted to assess shape mode associations with systolic function. PVR status correlated significantly with shape modes associated with right ventricular (RV) apical dilation and left ventricular (LV) dilation (*p* < 0.01), RV basal bulging and LV conicity (*p* < 0.05), and pulmonary valve dilation (*p* < 0.01). PVR status also correlated significantly with RV ejection fraction (*p* < 0.05) and correlated marginally with LV end-systolic volume index (*p* < 0.07). Shape modes discriminated subsequent PVR better than standard imaging indices (MCC = 0.49 and MCC = 0.28, respectively) and were significantly associated with RV and LV radial systolic strain. Biventricular shape modes discriminated differences between patients who did and did not require subsequent PVR better than standard imaging indices in current use. These regional features of cardiac morphology may provide insight into adaptive vs. maladaptive types of structural remodeling and point toward an improved quantitative, patient-specific assessment tool for clinical use.

## Introduction

Tetralogy of Fallot (TOF) is the most common cyanotic congenital heart disease (CHD) accounting for about 7–10% of all congenital cardiac malformations^1^. Current twenty-five-year mortality rates are ≤ 5%, but long-term sequelae in adult survivors with repaired tetralogy of Fallot (rTOF) lead to an acceleration in mortality thereafter^2^. Common late consequences of repair include residual pulmonary regurgitation (PR) and chronic right ventricular (RV) volume overload, which in turn are associated with exercise intolerance, arrhythmia, RV and/or left ventricular (LV) dysfunction, and a higher risk of sudden cardiac death^3,4^. The main strategy for preserving RV function relies on timely surgical or transcatheter pulmonary valve replacement (PVR), which has been shown to alleviate symptoms, normalize RV volumes, and improve RV function^5,6^. However, there continues to be disparity in the indications and timing for PVR^7–9^, which must strike a balance between being performed early enough to prevent irreversible adverse remodeling but late enough to reduce the number of re-interventions and potential surgical complications^10^.

Current indications for PVR rely on cardiovascular magnetic resonance (CMR) imaging, which is the gold standard for evaluation of RV volumes, severity of PR, and RV function^11,12^. The American College of Cardiology/American Heart Association, the Canadian Cardiovascular Society, and the European Society of Cardiology provide recommendations on indications for performing PVR^13–15^; though, they are largely qualitative in nature, recommending intervention in cases of moderate to severe RV dysfunction and/or moderate to severe RV enlargement. Quantitative indications for intervention are based on global measures of cardiac mass and volume^16,17^, e.g. RV end-diastolic volume index (EDVi) > 160 mL/m^2^ and/or RV end-systolic volume index (ESVi) > 80 mL/m^2^, but these thresholds are subject to institutional bias and lead to mixed outcomes, leaving the subject up for debate^18^. While these indications have been guided by several CMR-based imaging studies, the wealth of morphological and functional information available in CMR images has been under-utilized, not taking into account three-dimensional, regional analysis of geometry and function.

Several studies have shown that bulging of the RV outflow tract, dilation of the RV apex, dilation of the pulmonary valve annulus, and a more circular tricuspid valve are associated with adverse remodeling in rTOF, specifically in the presence of chronic PR^19,20^. Other studies of RV remodeling in rTOF have shown that a decrease in RV free wall curvature is associated with a decrease in regional RV function as measured by a lower area strain^21^. However, these prognostic markers are not captured by global measures of ventricular function or used in current clinical decision-making to indicate intervention.

The current study employed a statistical atlas-based approach to condense multi-dimensional, morphological data from CMR imaging into quantitative, interpretable markers of regional biventricular shape. These atlas-based biomarkers were assessed in their ability to discriminate differences in shape between patients who required subsequent PVR and those who did not, and the performance of these novel biomarkers was compared with standard imaging indices. The aim was to discover specific markers of cardiac morphology that could discriminate differences in adverse remodeling within the rTOF population, regarding referral for subsequent PVR, better than standard imaging indices.

## Methods

All methods were carried out in accordance with relevant guidelines and regulations.

### Study population

CMR images and associated clinical data from 84 rTOF patients were obtained retrospectively from the Cardiac Atlas Project (CAP) database^22^. The CAP is a worldwide consortium that facilitates clinical data sharing and development of computational methods for analysis of cardiac structure and function across several CHD cohorts (https://www.cardiacatlas.org). Deidentified datasets employed in this study were contributed from two clinical centers (Rady Children’s Hospital, San Diego, CA, US and The Center for Advanced Magnetic Resonance Imaging, Auckland, NZ) with approval from local institutional review boards via waiver of informed consent (UCSD IRB 201138 and HDEC 16/STH/248, respectively). Patients were divided into two groups, one in which patients were referred for PVR following imaging (PVR, n = 48) and one in which patients were not referred for PVR following imaging (No-PVR, n = 36), within a four-year period. Patients with previous PVR were excluded from the study. Patients with significant tricuspid regurgitation or only mild pulmonary insufficiency (PR fraction < 20%) by CMR were also excluded, such that the patients included in the study demonstrated an RV volume overload phenotype due strictly to moderate to severe PR. Out of the forty-eight patients included in the PVR cohort, twenty-six of them were symptomatic. The most common symptoms were tiredness, shortness of breath, and exercise intolerance, and in some cases, symptoms included chest pain or palpitations. While essentially all rTOF patients have a right bundle branch block (RBBB) pattern, none of the subjects in this study had significant degrees of atrial or ventricular ectopy or atrioventricular conduction disturbance. The decision to perform PVR at Rady Children’s Hospital was largely based on the criteria outlined by Tal Geva^18^, which includes thresholds for RV EDVi, RV ESVi, and RV and LV ejection fraction (EF), where the number of criteria that need to be met are reduced if the subject is symptomatic. The decision to perform PVR at The Center for Advanced Magnetic Resonance Imaging was based on the same threshold for RV EDVi as the Tal Geva criteria^18^, significantly reduced RV and LV EF, and considerations for the presence of symptoms. Summary characteristics of the study participants grouped by PVR status are shown in Table 1. Study participants from The Center for Advanced Magnetic Resonance Imaging were on average ten years older and included proportionally more female subjects than study participants from Rady Children’s Hospital. Participants from each of these centers were unequally represented in each clinical group contributing to significant differences in age and sex between these groups. Quantitative data regarding the degree of pulmonary valve stenosis were available for study participants from Rady Children’s Hospital San Diego. In this cohort, 90% of the patients demonstrated mild pulmonary valve stenosis as defined by a pulmonary valve peak gradient of less than 36 mmHg on an echo six months within the CMR exam date. In addition, based on clinical notes, none of the study participants from The Center for Advanced Magnetic Resonance Imaging had severe pulmonary valve stenosis.

**Table 1:** Summary characteristics grouped by PVR status.

Characteristic	PVR (n = 48)	No-PVR (n = 36)	*p* value
Sex (m/f)	35/13	17/19	0.016
Type of repair			0.003
Transannular patch	35	30	
Valve-sparing	2	6	
Conduit	11	0	
Age at repair (y)	0.3 (0.3–0.8)	1 (1–4)	< 0.001
Age at CMR (y)	12 (9–16)	20 (16–33)	< 0.001
Age at PVR (y)	14 (11–17)	–	
Time after repair to CMR (y)	12 (9–16)	19 (15–26)	< 0.001
Time after CMR to PVR (y)	0.9 (0.3–1.8)	–	
Height (cm)	156 (135–165)	163 (157–167)	0.006
Weight (kg)	49 ± 23	62 ± 18	0.008
BSA (m^2^)	1.4 ± 0.4	1.7 ± 0.3	0.002

Normally distributed data are reported as mean ± standard deviation or as median (interquartile range) otherwise.

*BSA* Body surface area, *CMR* Cardiovascular magnetic resonance, *PVR* Pulmonary valve replacement.

### CMR image acquisition and analysis

Each patient underwent functional CMR examination within the scope of standard clinical practice. CMR images were acquired using 1.5T MRI scanners, including Siemens Avanto (Siemens Medical Systems) and GE Discovery (GE Healthcare Systems). Two-dimensional cine images were acquired using steady-state free procession imaging and were prospectively or retrospectively gated during breath-hold. Short-axis slices were obtained parallel to the tricuspid annulus plane, spanning both ventricles from apex to base. Long-axis slices were obtained through all valve annuli in standard 4-chamber, 2-chamber, LV outflow tract, and RV outflow tract views. Typical imaging parameters included: repetition time 24-32 ms; echo time 1.1–1.5 ms; flip angle 70–80°; field of view 200–300 mm × 200–300 mm; spatial resolution 0.59–1.75 mm × 0.59–1.75 mm × 4-6 mm; and number of time frames 20–35.

Contours were drawn manually at end-diastole (ED) and end-systole (ES) on both short-axis and long-axis cine slices using Segment (Medviso). Papillary muscles were excluded from the endocardial contour segmentation. Mitral and tricuspid valve landmark points were defined from the intersection of the left and right atrial and ventricular contours delineated on the 4-chamber and 2-chamber long-axis images. Aortic valve landmark points were defined from the LV outflow tract images, and pulmonary valve landmark points were defined from the RV outflow tract images. When aortic and pulmonary leaflets were not visible, boundary points were defined by the change in appearance of the myocardial and vessel wall.

Antegrade and retrograde pulmonary flow measurements were obtained from two-dimensional phase-contrast (PC) imaging using commercially available software, including Argus Flow (Siemens Healthineers) and cvi42 (Circle Cardiovascular Imaging). PC analysis of antegrade and retrograde flows in the main artery was performed on a plane just below the pulmonary artery bifurcation and perpendicular to the axis of the pulmonary artery. Typical imaging parameters included: repetition time 4.8–5.0 ms; echo time 2.3–3.0 ms; flip angle 15–30°; field of view 169–315 mm × 300–420 mm; spatial resolution 1.4–2.0 mm × 1.4–2.0 mm × 5–8 mm; temporal resolution 37–41 ms; and acceleration factor 3. Scouts were used to set the velocity encoding.

CMR image analysis was performed by a senior researcher with more than ten years of cardiac image analysis experience. The expert analyst was blinded regarding the patient’s PVR status.

### Biventricular shape analysis

A biventricular subdivision surface template mesh was constructed including the LV endocardium, RV endocardium, epicardium, and all four valves as described previously^23^. The template mesh was automatically customized to each patient while correcting for breath-hold slice misregistration using an iterative registration algorithm. Valve locations were customized to the manual landmark points via landmark registration, and surfaces were customized to the manual contours via diffeomorphic non-rigid registration. Standard imaging indices, including LV and RV volumes and masses at ED and ES, were calculated by numerical integration of mesh volumes, which has previously shown good agreement with slice summation of manual contours^23,24^.

To build a biventricular atlas of ED shape, patient-specific ED surface points were first aligned to population mean ED surface points by a rigid registration. Following the alignment, principal component analysis (PCA) was used to evaluate the distribution of shape variation across the rTOF cohort. PCA is an unsupervised dimensional reduction technique that, in this case, condenses cardiac shape features into statistical Z-scores represented as orthogonal components (modes), ranked by the amount of shape variance they explain in the population, that quantify degrees of patient-specific shape difference from the population mean.

### Shape mode subset selection

A subset of biventricular shape modes was chosen from the rTOF atlas to create a feature set with the same number of variables as standard imaging indices calculated (10 variables). Modes that cumulatively explained greater than 95% of the variation in shape in the rTOF cohort were ranked for predicting PVR status using chi-square tests, which is a commonly used technique for univariate feature ranking for classification. The 10 modes with the highest predictive importance score were retained for multivariate associations with PVR status.

### Multivariate associations with PVR status

Multivariate regression models were constructed to quantify imaging index and biventricular shape associations with PVR status, while accounting for confounders. Sex, type of repair, time after repair, and body surface area (BSA) were included in the models to control for differences that exist in these characteristics between the PVR and No-PVR cohorts. When testing associations with imaging indices, the response variable was each imaging index. In these models, PVR status, sex, and type of repair were included as categorical predictors, and time after repair was included as a continuous predictor. When testing associations with biventricular shape, the response variable was the morphometric score for each shape mode. In these models, PVR status, sex, and type of repair were included as categorical predictors, and time after repair and BSA were included as continuous predictors. Imaging indices and shape modes that had the most significant associations with PVR status after accounting for confounders were retained for clustering to discriminate PVR status.

### Clustering analysis to discriminate PVR status

K-means clustering, an unsupervised clustering method, was employed to characterize the discriminatory power of imaging indices and biventricular shape (minimum two variables required in each feature set). Patients were divided into two clusters using the cosine distance metric to ignore the absolute sizes of measurements and only consider their relative sizes. The ability of imaging indices and shape modes to discriminate the PVR and No-PVR cohorts in these clusters was assessed through a matching matrix. The Matthews correlation coefficient (MCC) was used to measure the quality of clinical classification, which has been shown to be more reliable than accuracy and F_1_ score^25^.

### Geometric strain analysis

Geometric strain analysis was conducted to assess if differences in imaging indices and biventricular shape that are associated with PVR status are also associated with differences in systolic function. Longitudinal strain (LS), circumferential strain (CS), and radial strain (RS), which represent longitudinal shortening, shortening along the circular perimeter, and thickening of the wall, respectively, were calculated from geometric arc length changes between ED and ES using the Cauchy strain formula and have shown good agreement with myocardial strains calculated from myocardial tagging^26,27^. LS and CS were calculated from the model mesh, and RS was calculated using a modified version of the centerline method^28^ using the intersection between short-axis slices and the model mesh. The interventricular septum was included in the calculation for LV systolic strain. Univariate regression models were applied to quantify the association between LV and RV systolic strains and imaging indices and shape modes associated with PVR status.

### Statistical analysis

Statistical analysis was carried out using the SciPy Python library (https://www.scipy.org). Summary characteristics for the two groups are reported as mean ± standard deviation or as median (interquartile range), depending on the distribution, for continuous variables and as frequency for categorical variables. Normality was tested using Shapiro-Wilks. Differences between the two groups were tested using unpaired two-sample *t*-tests or Wilcoxon rank-sum tests, depending on the distribution, for continuous variables and Pearson’s chi-squared tests for categorical variables. All variables were normalized before regression and k-means clustering. Statistical associations in the regression analyses are denoted by *p* values with a significance level of 0.05.

### Ethics approval and consent to participate

Deidentified datasets employed in this study were contributed from two clinical centers (Rady Children’s Hospital, San Diego, CA, US and The Center for Advanced Magnetic Resonance Imaging, Auckland, NZ) with approval from local institutional review boards via waiver of informed consent (UCSD IRB 201138 and HDEC 16/STH/248, respectively).

## Results

### Standard imaging index features

Models were successfully customized to manual contours and landmark points for all patients. Ventricular volumes and masses were calculated by numerical integration of mesh volumes, and PR volumes were calculated from PC imaging. Together these measurements constituted the feature set of standard imaging indices used in subsequent analyses. Measurements were indexed to BSA. Summary imaging indices for the PVR and No-PVR cohorts are shown in Table 2.

**Table 2:** Summary imaging indices grouped by PVR status.

Imaging index	PVR (n = 48)	No-PVR (n = 36)	*p* value
LV EDVi (mL/m^2^)	79 ± 15	79 ± 15	0.923
LV ESVi (mL/m^2^)	43 ± 9	41 ± 10	0.341
LV EF (%)	46 ± 6	48 ± 6	0.057
LV Mi (g/m^2^)	75 ± 11	72 ± 14	0.341
RV EDVi (mL/m^2^)	140 ± 22	138 ± 32	0.723
RV ESVi (mL/m^2^)	87 ± 17	80 ± 23	0.110
RV EF (%)	37 ± 6	42 ± 7	0.001
RV Mi (g/m^2^)	42 ± 9	39 ± 7	0.080
PRVi (mL/m^2^)	27 ± 12	30 ± 17	0.363
PRF (%)	41 ± 10	40 ± 11	0.611

Data are reported as mean ± standard deviation.

*EDVi* End-diastolic volume index, *EF* Ejection fraction, *ESVi* End-systolic volume index, *LV* Left ventricular, *Mi* Mass index, *PRF* Pulmonary regurgitant fraction, *PRVi* Pulmonary regurgitant volume index, *RV* Right ventricular.

### Biventricular shape features

**Figure 1:**
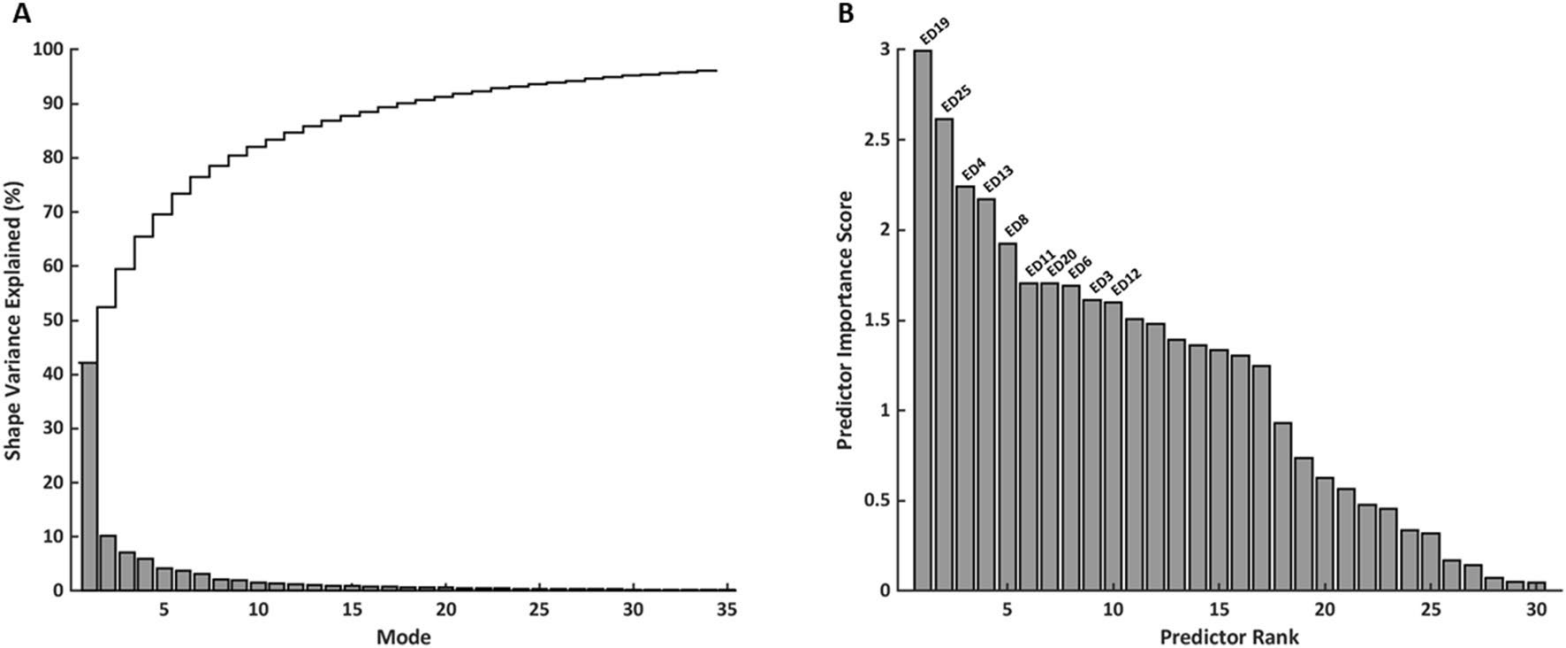
Shape mode subset selection from the rTOF atlas. (**A**) Shape variance explained (%) per mode (shaded bars) and cumulatively (solid line). (**B**) PVR status predictor rank and importance score for modes that explained greater than 95% of shape variation in the population. Labeled shape modes were retained for multivariate associations with PVR status.

PCA on anatomically co-registered models of 84 rTOF patient hearts yielded a statistical shape atlas comprised of 83 orthogonal components (modes). The percent of shape variance explained by each mode and cumulatively for the first 35 modes is shown in Fig. 1A. The first 30 modes explained greater than 95% of the shape variation in the population and were ranked for predicting PVR using chi-square tests. PVR status predictor ranks and importance scores for the first 30 modes are shown in Fig. 1B. The 10 modes with the highest predictive importance score are labeled and constituted the set of biventricular shape features used in subsequent analyses, equal to the number of standard imaging indices calculated.

### Imaging indices and shape modes associated with PVR status

Multivariate regression models were constructed to quantify imaging index and biventricular shape associations with PVR status while accounting for confounders including sex, type of repair, time after repair, and BSA. Summary results of imaging index and shape mode associations with PVR status are shown in Table 3.

**Table 3:** Summary results for imaging index and shape mode associations with PVR status after accounting for sex, type of repair, time after repair, and BSA.

Imaging index	*p* value	Shape mode	*p* value
**RV EF**	**0.043**	**ED4**	**0.002**
LV ESVi*	0.065	**ED25**	**0.003**
RV Mi	0.065	**ED6**	**0.022**
PRVi	0.113	ED20	0.115
LV EF	0.155	ED3	0.281
LV EDVi	0.226	ED19	0.337
LV Mi	0.376	ED8	0.593
RV ESVi	0.497	ED13	0.594
RV EDVi	0.667	ED11	0.695
PRF	0.837	ED12	0.704

Features in bold have significant associations with PVR status and were retained for clustering analysis.

*EDVi* End-diastolic volume index, *EF* Ejection fraction, *ESVi* End-systolic volume index, *LV* Left ventricular, *Mi* Mass index, *PRF* Pulmonary regurgitant fraction, *PRVi* Pulmonary regurgitant volume index, *RV* Right ventricular.

*LV ESVi was also retained for clustering analysis to meet the minimum two variables required for the imaging index feature set.

In the feature set of standard imaging indices, RV EF was significantly associated with PVR status, in which patients in the PVR cohort had lower RV EF than patients in the No-PVR cohort (Table 2). LV ESVi was the next most highly associated with PVR status, in which patients in the PVR cohort had higher LV ESVi than patients in the No-PVR cohort (Table 2). RV EF and LV ESVi were retained for clustering analysis from the imaging index feature set to meet the minimum two variables required.

**Figure 2:**
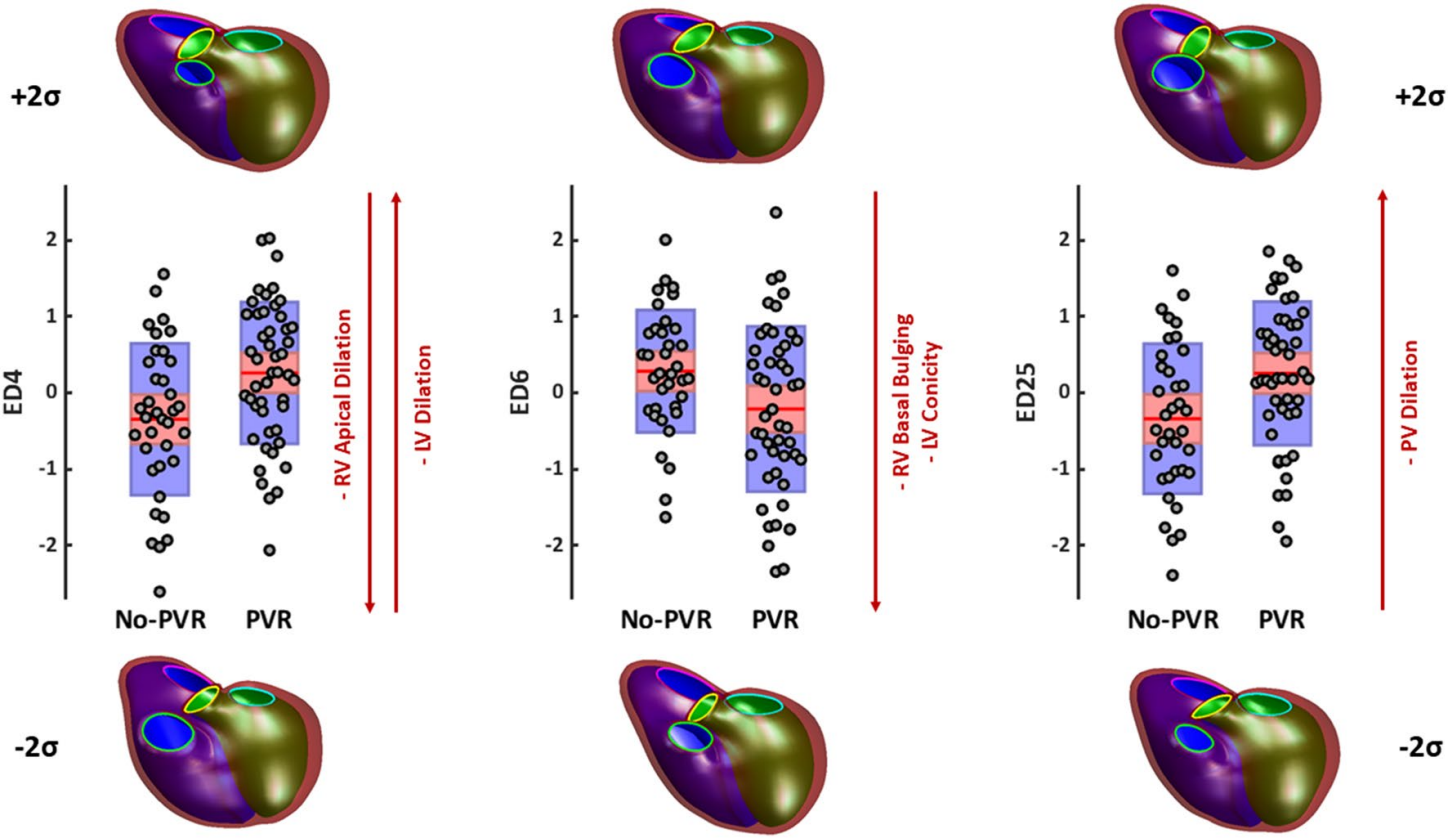
Shape modes that have significant associations with PVR status. Box plots show mean (red line), 95% confidence intervals of the mean (pink shaded bar), and one standard deviation of the mean (blue shaded bar) for Z-scores (gray circles). Models show mean ED shape plus two standard deviations (top) and minus two standard deviations (bottom) for each mode. The LV endocardial surface, RV endocardial surface, and epicardial surface are shown in green, purple, and maroon, respectively. The mitral, tricuspid, aortic, and pulmonary valves are shown in cyan, pink, yellow, and green, respectively. *LV* Left ventricular, *PV* Pulmonary valve, *RV* Right ventricular.

In the set of biventricular shape features, three shape modes were significantly associated with PVR status: ED4, ED6, and ED25. Shape changes along each of these modes are shown in Fig. 2 in addition to Z-scores for the PVR and No-PVR cohorts. ED4 appears to be a specific marker of opposing RV apical dilation and LV dilation; ED6 appears to be a specific marker of RV basal bulging and LV conicity; and ED25 appears to be a specific marker of pulmonary valve dilation. Patients in the PVR cohort demonstrated increased RV basal bulging, LV dilation, LV conicity, and pulmonary valve dilation, while patients in the No-PVR cohort demonstrated increased RV apical dilation. ED4, ED6, and ED25 were retained for clustering analysis from the shape mode feature set.

### Performance of imaging indices and shape modes in discriminating PVR status

**Figure 3:**
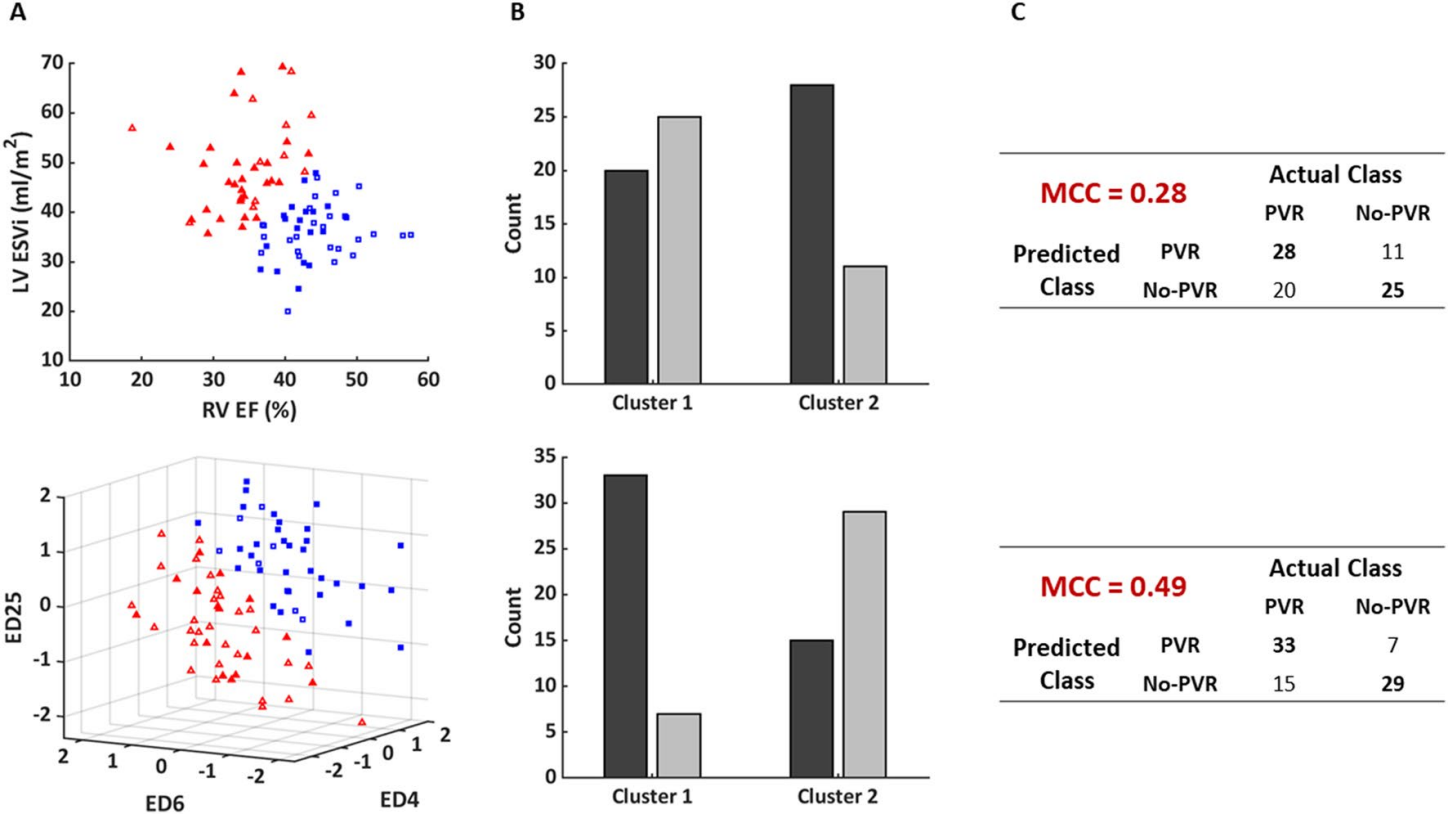
Summary clustering results depicting the ability of imaging indices (top) and shape modes (bottom) to discriminate PVR status. (**A**) K-means clustering using features with the most significant associations with PVR status with two clusters (blue squares: cluster 1; red triangles: cluster 2; filled: PVR; open: No-PVR). (**B**) Number of patients in the PVR (dark gray bars) and No-PVR (light gray bars) cohorts in each cluster. (**C**) Matching matrix and MCC performance metric for classifying patients by PVR status. *EF* Ejection fraction, *ESVi* End-systolic volume index, *LV* Left ventricular, *MCC* Matthews correlation coefficient, *RV* Right ventricular.

K-means clustering was employed to assess the ability of imaging indices and biventricular shape to discriminate between patients in the PVR and No-PVR cohorts. The results of the clustering analysis for each subset of features are shown in Fig. 3A. The number of patients in the PVR and No-PVR cohorts in each cluster for each subset of features are shown in Fig. 3B. From these results, a matching matrix was constructed and the MCC was calculated to assess the performance of clinical classification for each subset of features as shown in Fig. 3C, in which the predicted class was taken to be the dominant cohort in each cluster. The subset of shape modes discriminated patients in the PVR and No-PVR cohorts with much greater performance than the subset of standard imaging indices (MCC = 0.49 and MCC = 0.28, respectively).

### Imaging indices and shape modes associated with geometric strain

**Figure 4:**
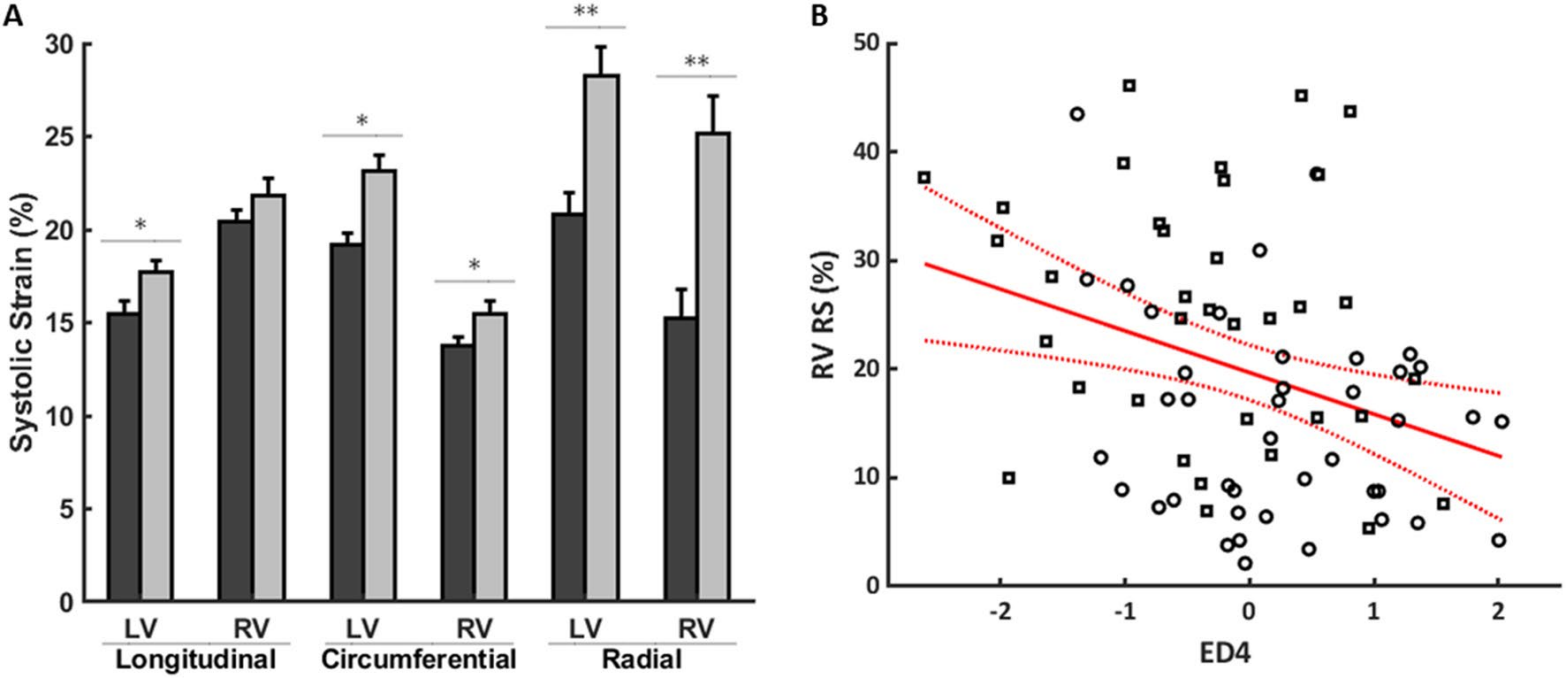
Geometric strain analysis. (**A**) Summary systolic strains (%) grouped by PVR status (PVR: dark gray bars; No-PVR: light gray bars). Data are reported as mean ± standard error. Systolic CS and LS are conventionally negative, but their absolute values are shown for simpler interpretation. Systolic strains that have significant associations with PVR status after accounting for sex, type of repair, time after repair, and BSA are denoted with symbols (*p < 0.05; **p < 0.01). (**B**) Univariate regression model between RV RS (%) and ED4 (black circles: PVR; black squares: No-PVR; red solid line: linear fit; red dotted lines: 95% confidence bounds; *p* < 0.01; *r* = 0.33). *LV* Left ventricular, *RS* Radial strain, *RV* Right ventricular.

Geometric strain analysis was used to assess if differences in imaging indices and biventricular shape that are associated with PVR status are also associated with differences in systolic function. Summary systolic strains for the PVR and No-PVR cohorts are shown in Fig. 4A. RV and LV RS were highly significantly associated with PVR status after accounting for confounders. For imaging indices, univariate regression analysis revealed that RV EF was highly significantly associated with LV LS, RV LS, LV CS, and RV CS (*p* < 0.0001) and significantly associated with LV RS (*p* < 0.05), where increased RV EF was associated with increased systolic strains. For shape modes, univariate regression analysis revealed that ED4 was highly significantly associated with RV RS as shown in Fig. 4B. ED6 and ED25 were also significantly associated with RV RS and LV RS, respectively (*p* < 0.05). A more positive ED6 and ED25 Z-score was associated with increased RV RS and decreased LV RS, respectively, and vice versa.

## Discussion

Adverse RV remodeling in rTOF has been well juxtaposed with RV morphology in reference healthy populations^29–31^; differences in RV remodeling within the rTOF population and how they determine referral for subsequent PVR, however, are not well characterized. In this cross-sectional retrospective study, an atlas-based biventricular shape analysis framework was used to quantify specific, regional shape differences associated with PVR status and compared with standard imaging indices in their ability to discriminate patients who did and did not undergo PVR.

### Standard imaging index features

Current guidelines^16–18^ recommend PVR in rTOF if progressive RV dilation results in RV EDVi > 160 mL/m^2^ and/or RV ESVi > 80 mL/m^2^; however, significant associations between these indices and subsequent PVR were not found. Interestingly, the degree of PR was also not significantly associated with PVR status, suggesting that the presence of volume overload alone was more important than current assessment of severity. This agrees with studies showing a compensatory increase in RV RS in response to PR and RV volume overload^32,33^. RV EF and LV ESVi had the highest associations with PVR status, although RV EF was the only imaging index with significant associations with PVR status. This was not unexpected as diminishing RV EF usually accompanies fairly rapid PVR (within a few weeks). These imaging indices were identified as preoperative risk factors for adverse outcomes post-PVR in the INDICATOR cohort study^34,35^, suggesting that these indices are not only associated with subsequent PVR but also are important determinants of long-term outcome.

### Biventricular shape features

Through biventricular shape analysis and geometric strain analysis, the present study shows that patients in the PVR cohort demonstrated increased RV basal bulging and pulmonary valve dilation, which was associated with reduced RV and LV RS, while patients in the No-PVR cohort demonstrated increased RV apical dilation, which was associated with increased RV RS. These specific, regional features of shape all contribute to RV dilation, but are not distinguished when relying solely on global ventricular measurements alone. While these shape changes agree with previously characterized features of adverse remodeling in the rTOF population as a whole^19–21^, this study suggests that RV basal bulging may be a form of maladaptive remodeling that leads to RV dysfunction (as in the PVR cohort), while RV apical dilation may be a form of adaptive remodeling that preserves RV function (as in the No-PVR cohort). Several studies have proposed mechanisms by which RV basal bulging may lead to RV dysfunction. One study found that increased RV basal bulging correlated with increased RV vorticity and that these changes in flow could induce abnormal fibrosis in these regions^36^. Another study found that RV basal bulging correlated with increased diastolic force parameters that resulted in decreased RV function^37^. Patients in the PVR cohort also demonstrated increased LV dilation and conicity, which have independently been shown to lead to LV dysfunction in patients with acute myocardial infarction^38^. This is also an important long-term follow-up issue for rTOF patients^39,40^.

By clustering analysis, biventricular shape modes discriminated PVR status better than standard imaging indices, despite the fact that these indices are currently the cornerstone of clinical evaluation for PVR. The clustering analysis was also repeated with the same imaging index and biventricular shape features with only patients that were asymptomatic (Supplement 1). The performance of the biventricular shape features in discriminating PVR status improved slightly (MCC = 0.51), while the performance of the imaging indices in discriminating PVR status decreased (MCC = 0.22). A matching matrix was also constructed where the predicted class was taken to be patients that did or did not meet criteria for PVR based on a single standard to avoid institutional bias regarding the decision to perform PVR and the actual class was taken to be whether or not the patient underwent PVR (Supplement 2). In this case, the set of criteria outlined by Tal Geva^18^ was employed, while specifically only accounting for RV EDVi, RV ESVi, and RV and LV EF thresholds and the presence of symptoms. The overall performance of these criteria in discriminating patients in the PVR and No-PVR cohorts was lower than biventricular shape features (MCC = 0.23). Upon closer inspection, these criteria were highly sensitive in discriminating referral for PVR (sensitivity = 0.94) but not very specific (specificity = 0.22), while biventricular shape features were not as sensitive in discriminating referral for PVR (sensitivity = 0.69) but much more specific (specificity = 0.81).

### Study limitations

Owing to the retrospective nature of this study, the clinical data available were heterogenous and limited in clinical indicators such as exercise capacity, rhythm disturbances, and functional scores. In the future, prospective studies should be conducted to assess biventricular shape mode relationships with differences in objective measures of outcome before and after PVR, rather than the decision to perform PVR itself, and supervised machine learning methods, such as decision trees or support vector machines, should be employed to fine-tune thresholds for atlas-based biomarkers that can serve as indications for PVR and give the desired specificity or sensitivity to maximize patient benefit. Another limitation of this study was the use of cross-sectional data. The temporal evolution of biventricular shape markers should be evaluated in longitudinal studies to get a better understanding of the time history of ventricular remodeling and how this relates to optimal timing of PVR. Finally, data employed in the study were contributed from multiple centers leading to selection bias that was unequally represented in each group. While confounders were accounted for, future analyses would benefit from an age and sex-matched comparison conducted at a single center.

## Conclusions

Specific biventricular shape modes in rTOF were able to discriminate differences between patients who did and did not require subsequent PVR better than standard imaging indices. These shape modes quantify regional features of cardiac morphology that may provide insight into adaptive vs. maladaptive types of structural remodeling that may be overlooked when relying solely on standard imaging indices alone. Routine clinical assessment of patients with rTOF using an atlas-based analysis of shape and function may reveal adverse effects of pathophysiologies over time, reduce qualitative observer and institutional measurement biases, and improve timing of interventions and patient prognosis.

## Supplementary Information

Supplementary Information.

## Data Availability

The deidentified patient data and shape models generated in this research are available on the Cardiac Atlas Project database (www.cardiacatlas.org).

## Abbreviations

BSA: Body surface area
CHD: Congenital heart disease
CMR: Cardiovascular magnetic resonance
CS: Circumferential strain
ED: End-diastole
EDVi: End-diastolic volume index
EF: Ejection fraction
ES: End-systole
ESVi: End-systolic volume index
LS: Longitudinal strain
LV: Left ventricular
Mi: Mass index
PR: Pulmonary regurgitation
PRF: Pulmonary regurgitant fraction
PRVi: Pulmonary regurgitant volume index
PV: Pulmonary valve
PVR: Pulmonary valve replacement
RS: Radial strain
rTOF: Repaired tetralogy of Fallot
RV: Right ventricular
TOF: Tetralogy of Fallot
