# Supplementary material for: Biventricular shape modes discriminate pulmonary valve replacement in tetralogy of Fallot better than imaging indices
Source: Sci Rep. 2023 Feb 9;13:2335. doi: 10.1038/s41598-023-28358-w (PMC9911768; doi:10.1038/s41598-023-28358-w)
Supplement: Supplementary file 1 — Supplementary Information. [file 41598_2023_28358_MOESM1_ESM.docx]

**Supplements**

**
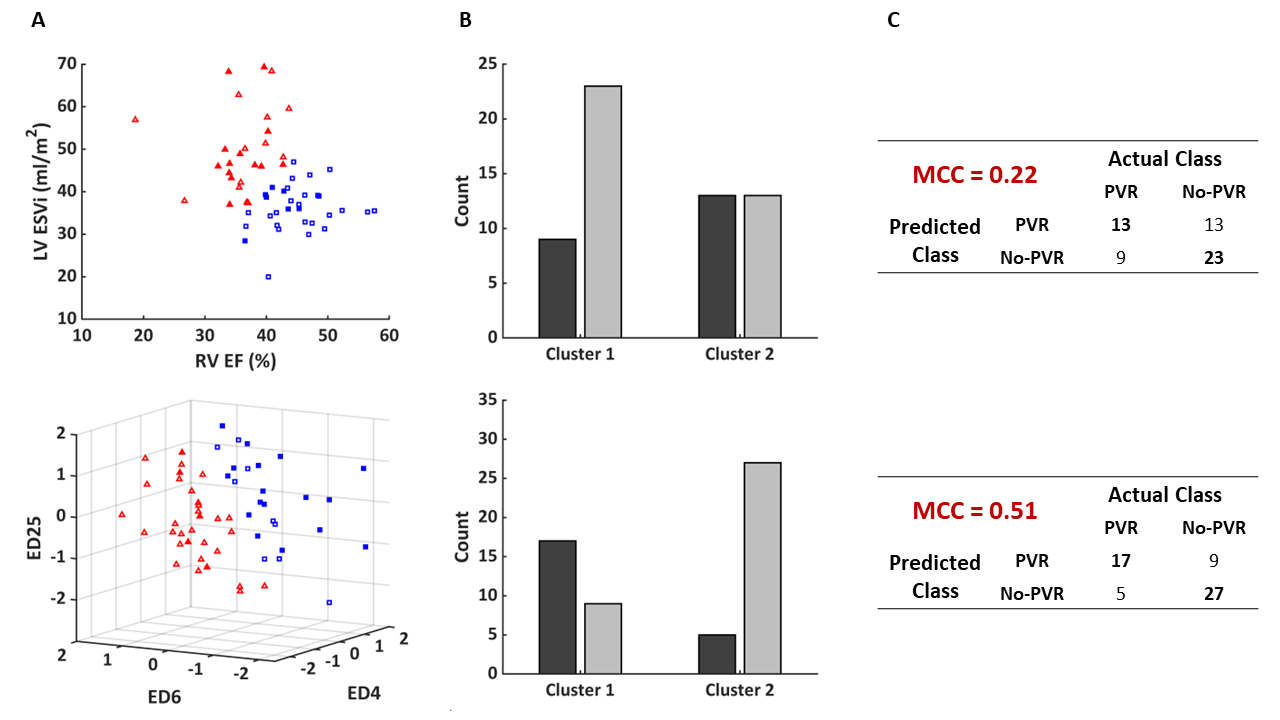
**

**Supplement 1. Summary clustering results depicting the ability of imaging indices (top) and shape modes (bottom) to discriminate PVR status for asymptomatic patients.** **A)** K-means clustering using features with the most significant associations with PVR status with two clusters (blue squares: cluster 1; red triangles: cluster 2; filled: PVR; open: No-PVR). **B)** Number of patients in the PVR (dark gray bars) and No-PVR (light gray bars) cohorts in each cluster. **C)** Matching matrix and MCC performance metric for classifying patients by PVR status. EF: ejection fraction; ESVi: end-systolic volume index; LV: left ventricular; MCC: Matthews correlation coefficient; RV: right ventricular.

**
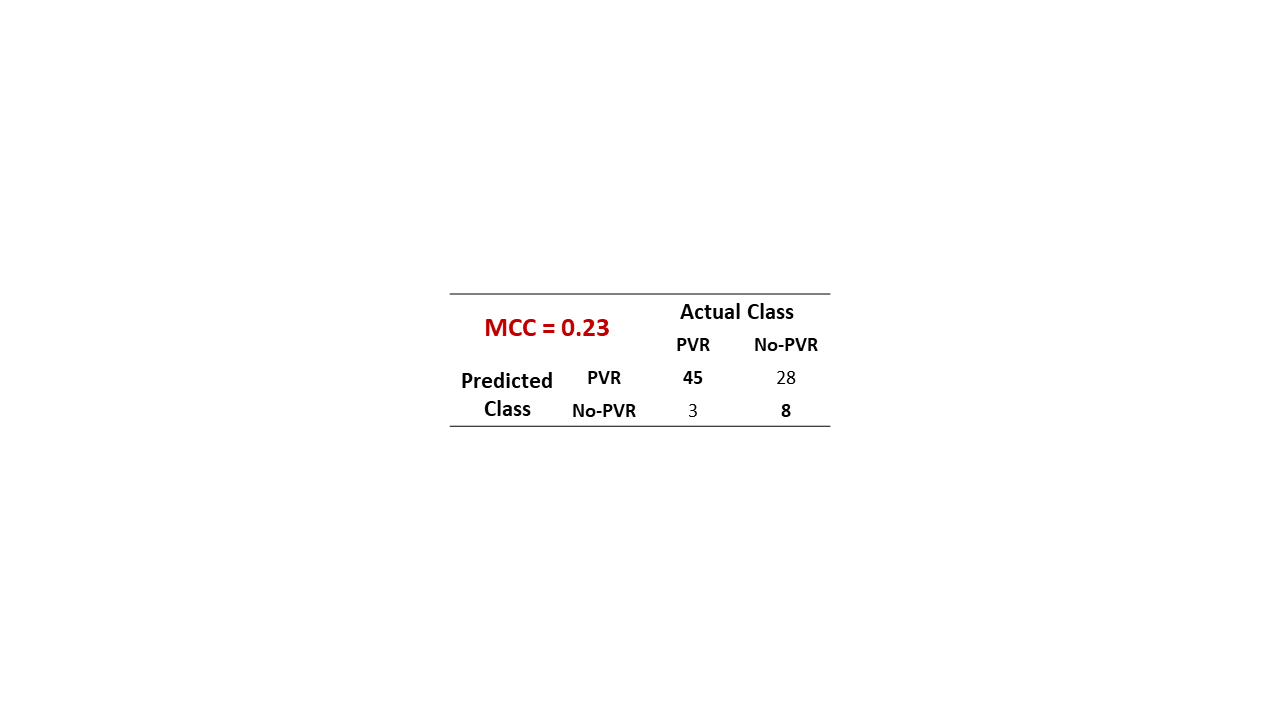
**

**Supplement 2. Matching matrix and MCC performance metric for classifying patients by PVR status based on volume and functional criteria outlined by Tal Geva.**
